# A Rare Case of Severe Haemolytic Anaemia Due to Folic Acid Deficiency in a Patient with Beta Thalassaemia Trait

**DOI:** 10.7759/cureus.79669

**Published:** 2025-02-26

**Authors:** Aleena Subair, Jeyanthy Rajkanna

**Affiliations:** 1 Internal Medicine, Peterborough City Hospital, Peterborough, GBR; 2 Diabetes and Endocrinology, Peterborough City Hospital, Peterborough, GBR

**Keywords:** beta thalassaemia trait, folic acid deficiency, haemolytic anaemia, homocysteine, non immune extravascular haemolysis

## Abstract

Severe haemolytic anaemia is a rare and often overlooked presentation of folic acid deficiency. Here we describe a case of severe haemolytic anaemia secondary to folic acid deficiency in a patient with beta thalassaemia trait. A 20-year-old young woman of Pakistani origin, previously independent and active with no significant past medical history, presented with vertigo, dizziness, tiredness, and muscle stiffness. On admission, she had a haemoglobin level of 23 grams/L, folic acid level <1.3 micrograms/L with evidence of haemolysis. Her peripheral blood film was consistent with severe haematinic deficiency. Following diagnosis of severe normocytic anaemia and non-immune extravascular haemolysis secondary to folate deficiency, she received blood transfusions and high-dose oral folic acid for three months. On follow-up, haemoglobin level had improved to 104 grams/L, folate levels were replenished, and she had resumed her normal life. She was incidentally found to have beta thalassaemia trait which explained the persistent mild anaemia. Hence, folic acid deficiency when significant may lead to severe haemolytic anaemia but shows excellent haematological response to treatment with high dose oral folic acid.

## Introduction

Folic acid, also known as vitamin B9 or folacin, is an essential water-soluble vitamin naturally found in dark green leafy vegetables, beans, peanuts, sunflower seeds, liver, eggs, seafood and fortified food; hence, its deficiency is rare. Folate, like vitamin B12, is a provider of one-carbon residues for DNA and RNA synthesis [1]. In situations like pregnancy, alcoholism, malabsorption, coeliac disease, intestinal surgeries and genetic variants, the risk of deficiency is increased. Drugs such as methotrexate, phenytoin, sulfasalazine, and trimethoprim can antagonise folate utilisation and inhibit its absorption or conversion to its active form, resulting in folate deficiency [1]. The prevalence of folate deficiency amongst adults and children is no more than 5% taking into account the World Health Organisation (WHO) clinical threshold of folate deficiency defined as serum folate less than 7 nano mol/L [2]. Here we present the case of a 20-year-old female with severe haemolytic anaemia secondary to significant folate deficiency and incidental diagnosis of beta thalassemia trait.

This article was originally presented as a poster at the Royal College of Physicians regional poster competition 2023 for the Eastern region on 2nd November 2023.

## Case presentation

A 20-year-old young woman presented to our emergency department twice over a period of two months with a history of progressive vertigo, dizziness, tiredness, and muscle stiffness. On first attendance, she was diagnosed as probable viral labyrinthitis and managed conservatively. She was also found to have mild anaemia with a haemoglobin level of 110 grams/L. Six weeks later, she consulted primary care with complaints of stiffness of leg muscles for which she was prescribed ibuprofen and was advised to mobilise as pain allows. An enthusiastic university student, fully independent and active until then, became increasingly bed-bound over two months due to unsteadiness and fear of falling. Her family assisted her with personal care and meals were served in bed. A further two weeks later, she presented to our emergency department with progression of symptoms following a spike in temperature and an episode of vomiting. Her initial observations showed respiratory rate of 29 per minute, heart rate of 135 per minute, temperature of 36.5 degrees Celsius and blood pressure of 125/66 mmHg. On general examination, she was afebrile and appeared pale with tachypnoea and tachycardia. Her systemic examination was normal. There were no signs of jaundice, hepatomegaly or splenomegaly. As part of the full history taking, it was mentioned that her family was originally from Pakistan, but she was born and brought up in the United Kingdom. She has three sisters and one brother who are all healthy, with no reported health conditions. She has no significant past medical or medication history. However, there is a family history of paternal grandmother requiring recurrent blood transfusions from childhood but they were unaware of the diagnosis. She is a non-vegetarian, non-smoker, and non-drinker but her mother reported that she was a fastidious eater since childhood avoiding vegetables when possible. She has a 28-day menstrual cycle with five days of menstruation and no history of passing clots.

Investigations

**Figure 1:**
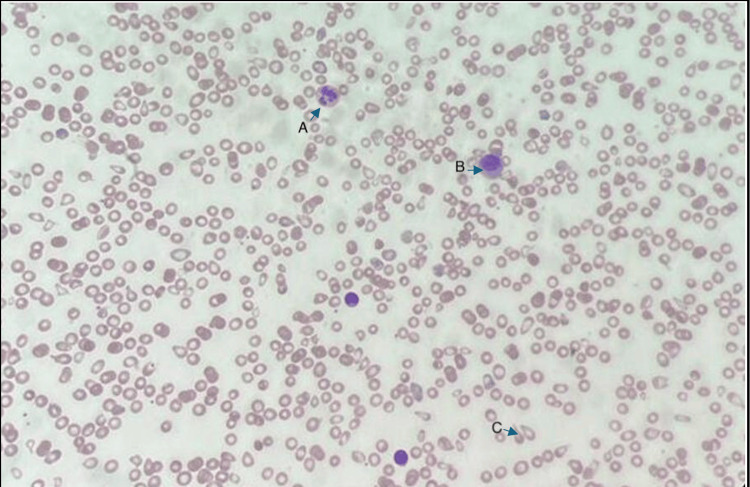
Peripheral blood smear of patient on admission A- hypersegmented neutrophils, B- reticulocytes, C- anisopoikilocytes

**Figure 2:**
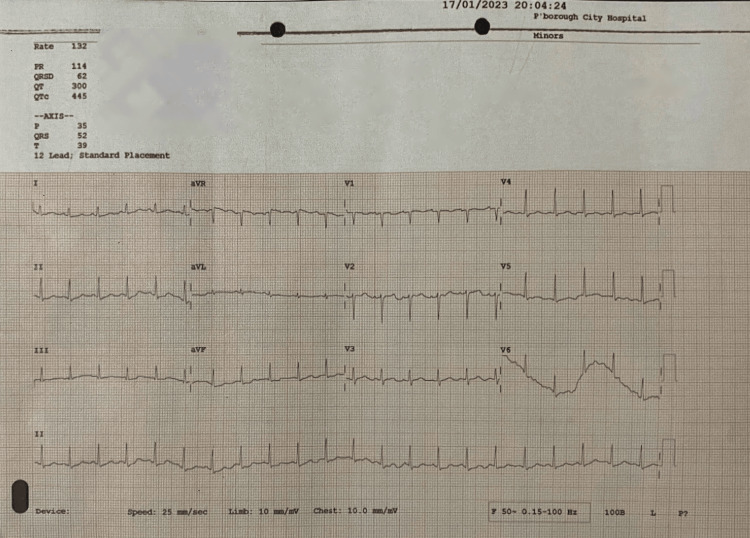
Electrocardiogram (ECG) on admission showing sinus tachycardia with heart rate 132 beats per minute

Laboratory investigations (Table 1) showed severe microcytic anaemia with evidence of haemolysis. However, red cell fragmentation was not a prominent feature in peripheral blood smear (Figure 1). Furthermore, direct Coomb’s test was negative suggestive of non-immune extravascular haemolysis. Cytomegalovirus serology indicative of past infection was considered insignificant. Electrocardiogram done on admission showed sinus tachycardia (Figure 2) and an abdominal ultrasonography undertaken to investigate hepatosplenomegaly was reported as normal.

**Table 1:** Laboratory investigations on admission and at monthly follow-ups thereafter. MCV- Mean corpuscular volume, PNH- Paroxysmal nocturnal haemoglobinuria, G6PD- Glucose-6-phosphate dehydrogenase

Investigations	On admission	Time of follow up since discharge			Normal range
		At 4 weeks	At 8 weeks	At 12 weeks	
Haemoglobin	23	104	110	108	115-165 grams/Litre
White blood cells	11.1	5.9	10.3	9.2	x10^9^/L
Haematocrit	0.079	0.319	0.358	0.345	0.360-0.460
MCV	89.8	71.1	59.8	54.9	80-100 femtolitre
Platelet	84	331	370	342	150-400 x10^9^/Litre
Reticulocyte count	98	14	23	86	23-81 x10^9^/Litre
Folate	<1.3	>19.5	>19.5	10	>3 microgram/Litre
Ferritin	208	97	74		30-400 microgram/Litre
Iron	47.7	5.9	9.6		10-28micromol/Litre
Vitamin B12	243	215	302	242	197 -771 picograms/millilitre
Total bilirubin	50	15	5	4	5.1 -17 micromol/Litre
Unconjugated bilirubin	24	8			3.4 -12 micromol/Litre
Haptoglobin	<0.1	0.2			0.4 -2.8 grams/Litre
Lactate dehydrogenase	1994	217			<250 units/Litre
Direct Coomb’s test	Negative				
PNH screen	Negative				
Sickle cell screen	Negative				
Coeliac screen	Negative				
G6PD level	Normal				
Pyruvate kinase	Normal				
Beta thalassaemia trait		Positive			

Treatment

Initial venous blood gas showed lactate of 11 milli mol/L, bicarbonate of 14 milli mol/L and a normal pH of 7.361, confirming compensated metabolic lactic acidosis due to tissue hypoxia. She received three units of packed red blood cells transfusion for acutely severe symptomatic anaemia, following which her lactate levels improved to 3.4 milli mol/L. 48 hours into admission, her lactate became normal with initial resuscitation and she was commenced on oral folic acid at a high dose of 10 mg once daily. After discussions with haematology and gastroenterology team, it was established that the most probable cause of her anaemia would be a haematological cause such as significant folic acid deficiency. Hence, a multidisciplinary team decision was made to continue the current high dose of folic acid for three months with four weekly haematology clinic follow-ups and then consider reducing the dose to 5 mg once daily orally depending on clinical response.

Outcome and follow-up

She recovered sufficiently from her symptoms following blood transfusions and was discharged with her first outpatient follow-up in four weeks. By her first follow-up in haematology clinic, which was four weeks after discharge, she had resumed her normal life including university and a part-time job. Laboratory investigation showed a persistent mild anaemia which was attributed to beta thalassaemia trait incidentally diagnosed on haemoglobinopathy screen. Her haemoglobin A2 (HbA2) was 4.5% (normal range is 1.8-3.5%) and foetal haemoglobin (HbF) was 1.9% (normal range is <2.0%), which is diagnostic for beta thalassaemia trait. She was followed up again at eight weeks and 12 weeks from discharge with full blood count and haematinics repeated in the clinic (Table 1). As her haemoglobin and folate levels remained stable at her final follow-up at 12 weeks from discharge, it was decided to reduce the folic acid dose to 5 mg once daily orally to continue for the next three months and she was discharged to her general practitioner for routine review and folic acid dose titration.

**Figure 3:**
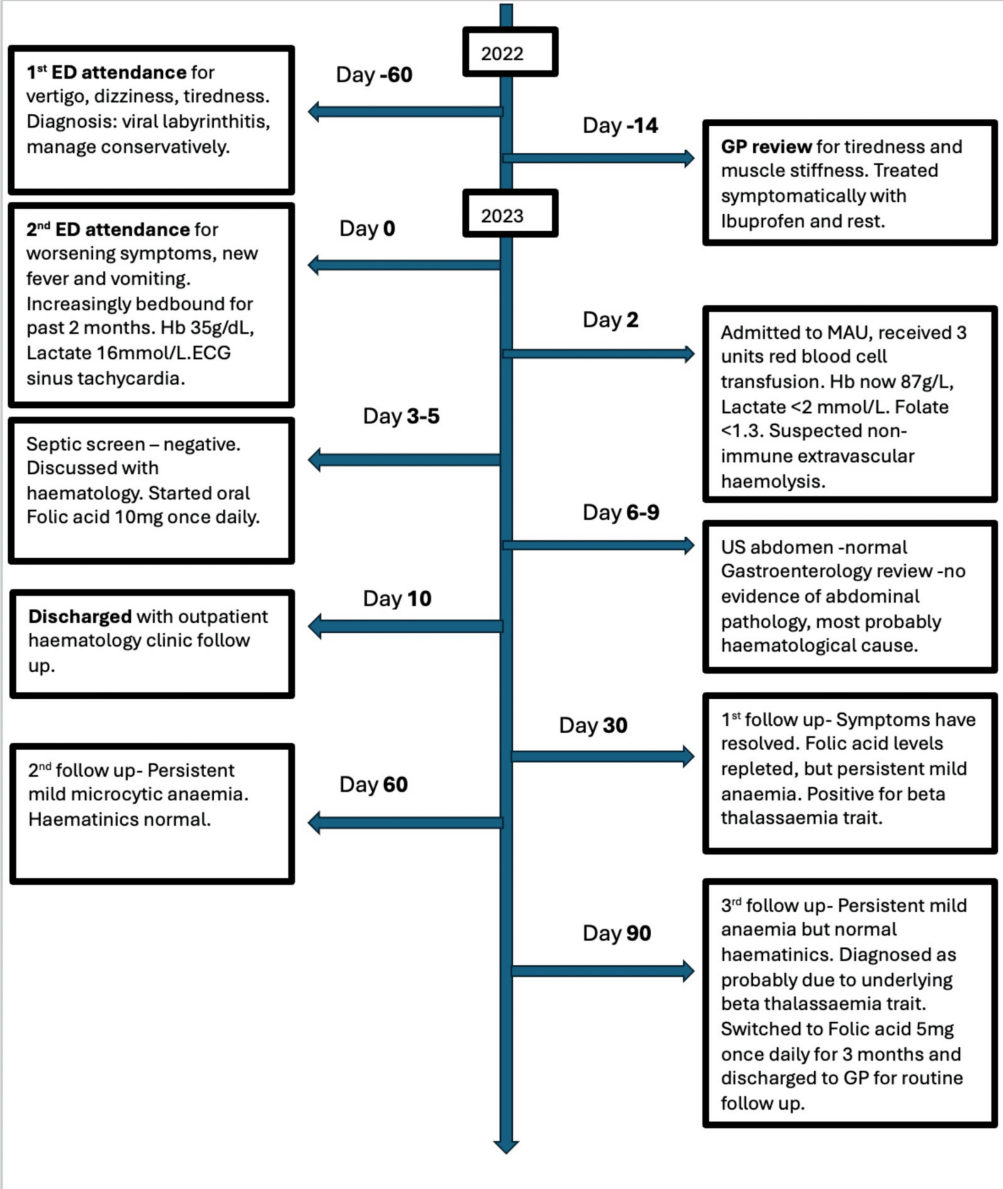
Timeline outlining key events and interventions. ED- Emergency Department, GP- general practitioner, MAU- Medical Assessment Unit, ECG- electrocardiogram, US- ultrasound, g/dL- grams/decilitre, g/L- grams/litre

Below is a timeline illustrating key events and interventions undertaken (Figure 3).

## Discussion

Folate is essential for erythropoiesis and foetal neural tube development [1]. In a state of deficiency, there is premature release of erythroblasts into circulation resulting in macrocytic anaemia; however, haemolysis is an uncommon presentation. Haemolytic anaemia occurs due to destruction of red blood cells either in circulation (intravascular) or outside the circulation (extravascular). The aetiology is classified into immune causes such as autoimmune haemolytic anaemia, drugs, delayed haemolytic transfusion reactions and non-immune causes such as haemoglobinopathies (includes sickle cell disease, thalassaemia) and microangiopathic haemolytic anaemia (such as disseminated intravascular coagulation) [3]. In our patient, we presume that her significant folate deficiency was primarily due to dietary deficiency as other differentials were excluded with history and investigations. We did not test for parvovirus B19 and mycoplasma due to low clinical suspicion. A bone marrow biopsy was not undertaken as our patient improved with initial treatment.

In a quest to understand the mechanism of haemolysis, a literature review was undertaken and we found the following remarkable case reports. Zhang Q et al. reported an exceedingly rare presentation of severe folate deficiency-induced non-immune haemolytic anaemia [4]. A 39-year-old young man with a history of alcohol abuse presented with acute on chronic abdominal pain and jaundice [4]. His liver enzymes and abdominal imaging were unremarkable but he had severe folic acid deficiency [4]. It was proposed that folate deficiency caused a non-immune brisk haemolysis with compensatory haemolysis [4].

Pratap T et al. reported a rare case of severe anaemia due to vitamin B12 and folate deficiency with non-immune intramedullary haemolysis [5]. A 40-year-old Caucasian woman with no significant past medical history presented with breathlessness, bilateral leg swelling and reduced exercise tolerance [5]. She was severely anaemic due to folate and vitamin B12 deficiency resulting in non-immune intramedullary haemolysis [5].

Another proposed mechanism was that elevated homocysteine levels found in folic acid deficiency could have pro-oxidant properties causing endothelial damage with subsequent microangiopathy [6,7]. Both vitamin B12 and folate are responsible for the re-methylation process that converts homocysteine to methionine [4,8]. A lack of either of them will lead to ineffective haematopoiesis with subsequent accumulation of homocysteine resulting in nonimmune haemolysis [4,8].

Furthermore, we expected a microcytic anaemia since our patient had beta thalassaemia trait, but she had a normocytic anaemia which became microcytic once her folate stores were restored. Castaldi et al. reported that plasma and red cell folate mean contents have been found to be significantly lower in 41 symptom-free β-thalassaemia heterozygotes than in 21 controls [9]. Such a decrease is considered an effect of increased folate utilization caused by the enhanced erythropoiesis [9]. The frequent finding of reduced red cell folate contents suggests that further folate imbalance might lead to clinically significant degrees of folate deficiency [9].

## Conclusions

In conclusion, severe anaemia and haemolysis such as in this case are rare clinical presentations of folic acid deficiency with limited published reports. Severe folic acid deficiency may result from nutritional deficiency as in our patient's case. As good practice, it is essential to obtain a detailed history and examination, investigate and exclude other causes of anaemia and haemolysis in any patient presenting with haemolytic anaemia. Additionally, haematinics, haemoglobinopathy screen, initial homogentisic acid level and bone marrow biopsy may be considered prior to initiation of treatment.
